# Pain and Heart Rate Variability in Caregivers of People with Dementia and Non‐Caregivers

**DOI:** 10.1002/alz70856_107597

**Published:** 2026-01-09

**Authors:** Christine McClure, Nora Mattek, Zachary T Beattie, Jeffrey A Kaye, Allison Lindauer

**Affiliations:** ^1^ Oregon Health & Science University, Portland, OR, USA; ^2^ Oregon Center for Aging & Technology (ORCATECH), Portland, OR, USA; ^3^ NIA‐Layton Aging & Alzheimer's Disease Research Center, Portland, OR, USA; ^4^ Oregon Health & Science University, Oregon Center for Aging & Technology (ORCATECH), Portland, OR, USA; ^5^ NIA‐Layton Aging & Alzheimer's Disease Research Center, Oregon Health & Science University, Portland, OR, USA; ^6^ Oregon Health and Sciences University, Portland, OR, USA; ^7^ Oregon Alzheimer's Disease Research Center, Portland, OR, USA

## Abstract

**Background:**

It is estimated that around 50% of caregivers experience chronic pain and caregiver burden and at least a quarter experience pain that limits daily activities. Heart rate variability has been utilized as an objective measure of stress and caregiver burden. While most research to date has focused on negative aspects of living with dementia, little research has investigated differences in pain and heart rate variability in caregivers as compared to non‐caregivers.

**Method:**

Weekly online reports of pain intensity and pain interference were collected on visual analog scales and heart rate variability total scores were collected nightly via Emfit bed mats for three months in the Oregon Center for Aging & Technology (ORCATECH), a study using unobtrusive remote home sensing and monitoring of health metrics in older adults.

**Result:**

The sample consisted of older adult twelve caregivers and twelve non‐caregivers matched on age, education, and bed‐sharing habits. Mean age was 73.7 ± 6.3 years, mean education was 15.4 ± 2.4 years, and 75% were female. Over three months, non‐caregivers showed higher (better) heart rate variability scores than caregivers (61.7 versus 49.7, respectively, *p* = 0.16). Neither pain intensity (caregivers = 2.1; controls = 1.5; *p* = 0.78) nor pain interference (caregivers = 1.7; controls = 1.6; *p* = 0.71) differed between groups.

**Conclusion:**

While this study was not powered to identify meaningful differences, it demonstrates the feasibility of using an objective digital assessment of caregiver burden (heart rate variability) in relation to caregiver pain. The findings could facilitate greater understanding of the role pain plays in caregiver burden.

